# Synovial sarcoma of the buttocks presenting with a non-healing wound and rapid progression after local resection: a case report

**DOI:** 10.1186/1477-7819-10-125

**Published:** 2012-06-28

**Authors:** Hai-Yan Zhang, Ye Feng, Zhuo Zhang, Ge Gao, Ji-Sheng Zhao

**Affiliations:** 1 Department of Gastrointestinal Surgery, China-Japan Union Hospital (The Third Clinical Hospital), Norman Bethune College of Medicine, Jilin University, Changchun, China; 2 Department of Gastrointestinal Surgery, China-Japan Union Hospital (The Third Clinical Hospital), Norman Bethune College of Medicine, Jilin University, No. 126 Xiantai Avenue, Changchun, 130033, China

**Keywords:** Non-healing wound, rapid progression, synovial sarcoma

## Abstract

Synovial sarcoma is a malignant mesenchymal neoplasm that is frequently misdiagnosed as a benign condition because of its small size, slow growth, and well-delineated appearance. Rapid spread and early death occur rarely. Here we report a case of synovial sarcoma of the buttocks presenting with a non-healing wound and rapid progression after local resection in a 23-year-old woman. She initially found a slightly painful subcutaneous mass in the left buttock and underwent local excision. Postoperatively, she developed a non-healing wound that did not respond to conventional antibiotic therapy and local wound care, and pitting edema of the lower extremities. A magnetic resonance imaging scan revealed a large heterogeneous, irregular mass in the buttocks with regional lymph node involvement. Histological and immunohistochemical analyses suggested the diagnosis of a poorly differentiated synovial sarcoma. Her condition deteriorated dramatically shortly thereafter; she developed systemic edema and died of respiratory failure. This case suggests that synovial sarcoma may be fatal within months of recognition if improperly managed and stresses the importance of adequate pre-surgical evaluation and postoperative pathological analysis in the management of a subcutaneous mass.

## Background

Synovial sarcoma is a malignant mesenchymal neoplasm that constitutes 8 to 10% of all sarcomas and has an annual incidence of 2.5 per 100,000. It mainly affects adults in the third to fifth decades of life [1] and usually arises in the extremities, especially the lower extremities around the knees [2]. This malignancy has a highly variable course. It is frequently misdiagnosed as a benign condition as a consequence of its small size, slow growth and well-delineated appearance. Overall, survival rates are 23.5 to 64% at 5 years and 11.2 to 34% at 10 years [3]. Rapid spread and early death occur infrequently. In addition, the development of non-healing wounds following local resection is rarely seen in patients with a synovial sarcoma. Here, we report a case of synovial sarcoma of the buttock that presented with a non-healing wound and rapid progression after local resection.

## Case presentation

A 23-year-old woman was admitted to our hospital due to the development of a non-healing postoperative wound for approximately 4 months. In January 2011, she found a slightly painful subcutaneous mass that measured 1 × 1.5 cm in the left buttock and underwent local excision at a local hospital. On postoperative day 2, she developed bleeding from the wound and received hemostatic interventions. However, the wound remained unhealed and continued to produce purulent exudates, with no obvious improvement after repeated dressing changes. On March 26^th^, 2011, the patient was referred to another hospital, where she underwent surgical debridement. Postoperatively, the wound deteriorated rapidly and developed a cauliflower-like growth. Meanwhile, she developed pitting edema of the lower extremities. Her general condition worsened.

**Figure 1:**
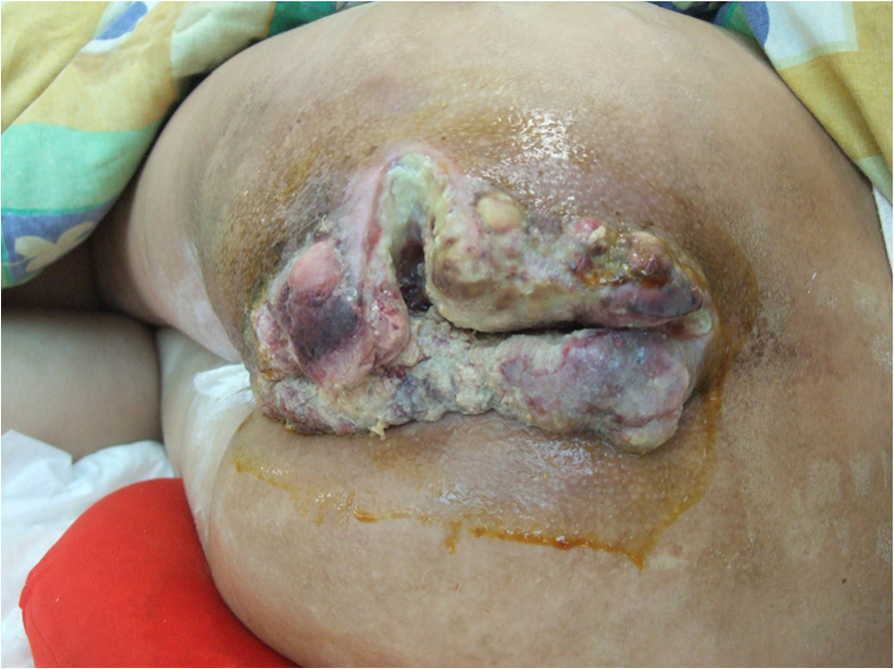
A clinical image showing a cauliflower-like lesion that measured 15 × 20 cm over the buttock region.

On April 21^st^, 2011, she was referred to our hospital. At the time of presentation, her general condition was poor and she had severe pallor. Her body posture was constrained and she could only sleep on the right side. There was obvious pitting edema of the bilateral lower extremities (especially the left side) and the lower abdomen. A T-shaped incision was noted on her left buttock. The cauliflower-like lesion measured 15 × 20 cm and was sufficiently deep to reach the ischial tuberosity, with a large amount of purulent discharge (Figure 1). Multiple inguinal lymph nodes were enlarged, hard, painful palpable, and fused together. Laboratory investigations revealed a red blood cell count of 1.39 × 10^12^/L, hemoglobin was 31 g/L, white blood cell count was 16.54 × 10^9^/L, neutrophil percentage was 83.6%, platelet count was 322 × 10^9^/L, and the albumin level was 14.3 g/L.

**Figure 2:**
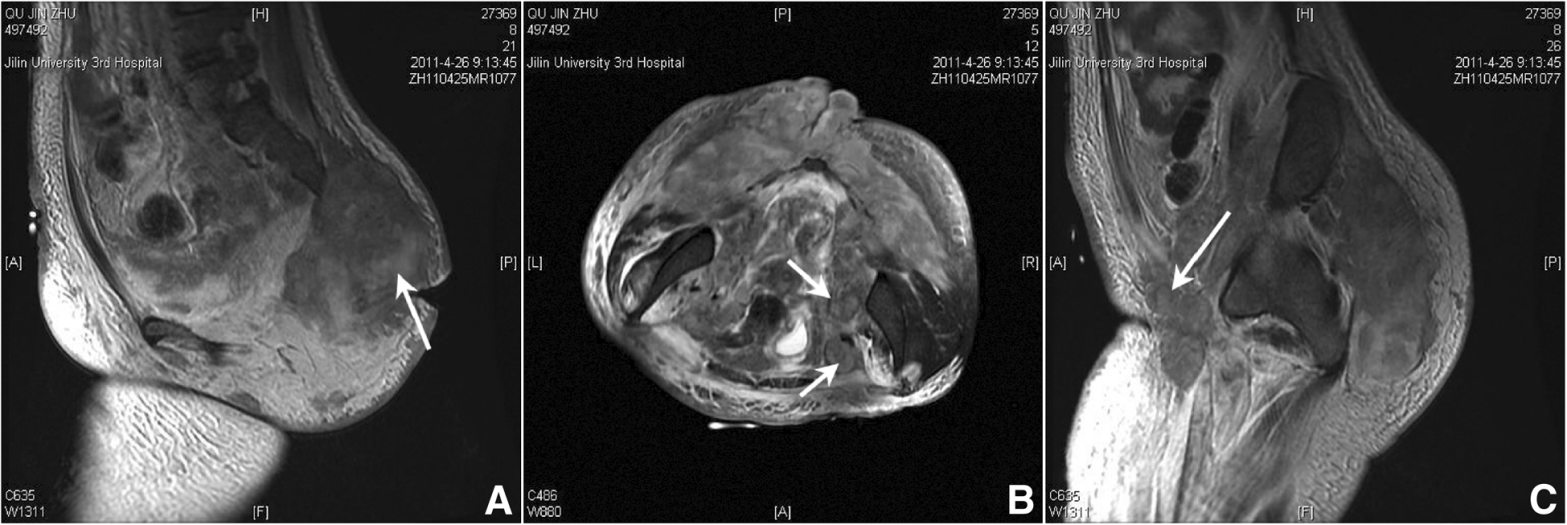
Magnetic resonance images revealing a large heterogeneous, irregular mass in the buttocks (A, arrow) and involvement of the pelvic (B, arrows) and inguinal lymph nodes (C, arrow).

**Figure 3:**
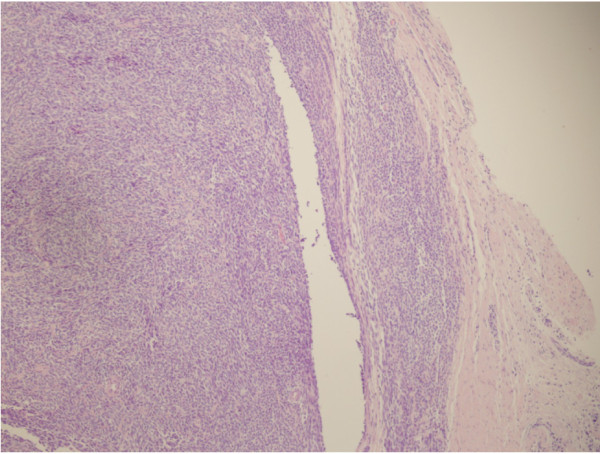
**H&E staining of the synovial sarcoma.** The tumor is characterized by the presence of numerous small round cells with a high nucleocytoplasmic ratio. Magnification: 100×.

**Figure 4:**
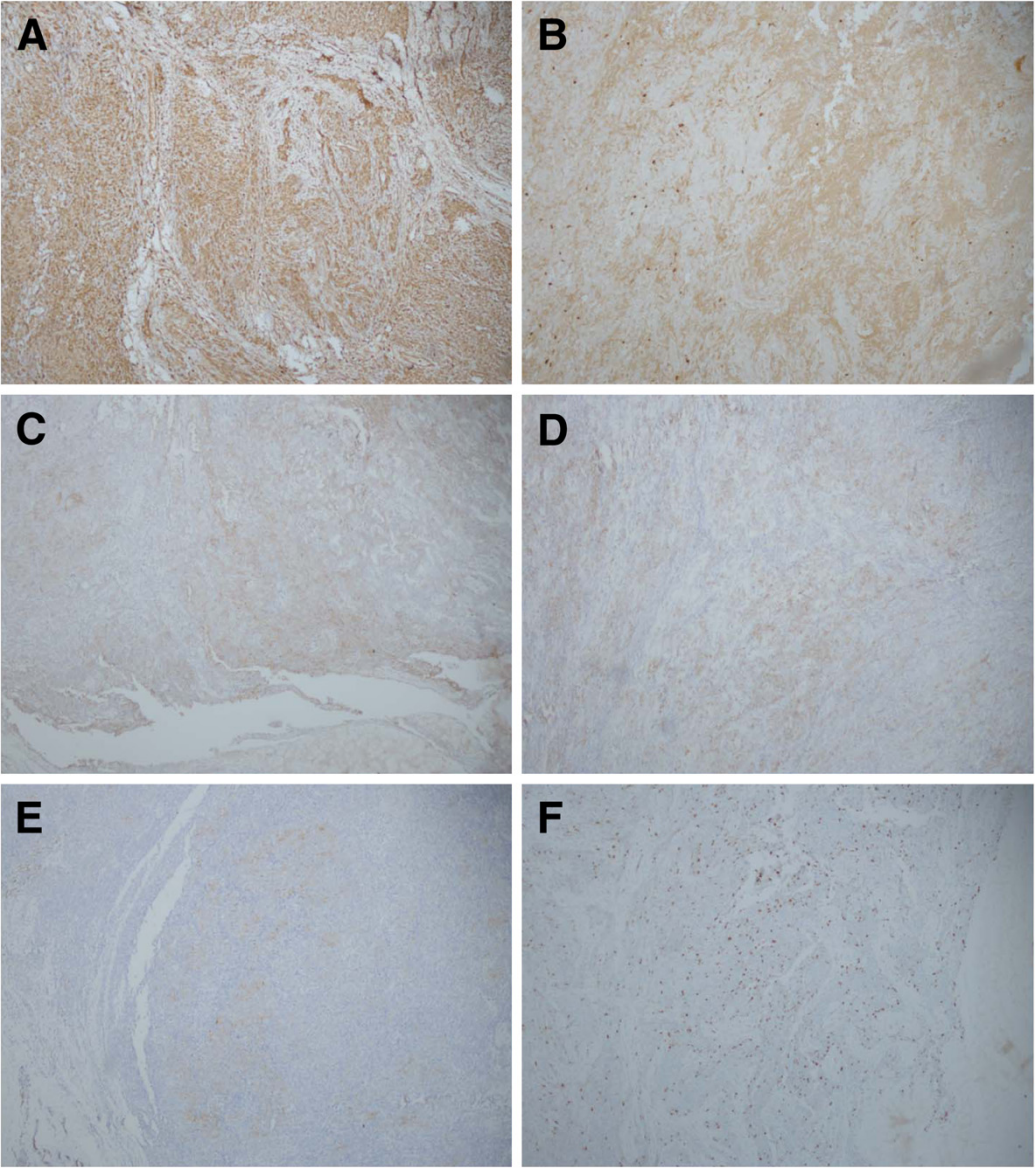
**Immunohistochemically stained sections indicating that the tumor cells are strongly positive for vimentin (A) and calretinin (B), moderately positive for CD99 (C) and epithelial membrane antigen (D), and partially positive for cytokeratin (E) and Ki67 (F).** Magnification: 100×.

A magnetic resonance imaging (MRI) scan was performed, which revealed a large heterogeneous, irregular mass in the buttocks, buttock ulceration, and multiple enlarged pelvic and inguinal lymph nodes that were fused together (Figure 2). An initial diagnosis of soft tissue sarcoma with lymph node metastasis was made. Small biopsy specimens were then taken and subjected to pathological examination, which revealed focal necrosis and tumor components. Numerous small round cells, with mostly oval nuclei and scarce cytoplasm, were seen (Figure 3). Immunohistochemically, the tumor cells were strongly positive for vimentin and calretinin (Figure 4A,B), moderately positive for CD99 and epithelial membrane antigen (EMA) (Figure 4C,D), partially positive for cytokeratin and Ki67 (Figure 4E,F), but negative for actin, S-100, desmin, and HMB45. Based on these results, a poorly differentiated synovial sarcoma was diagnosed.

As the patient developed severe anemia, hypoproteinemia and severe edema, she was treated with a blood transfusion, fluid infusion, supplementation with albumin and plasma, and regular dressing changes to maintain stable vital signs. However, her condition deteriorated dramatically and she rapidly developed systemic edema. As a result, she developed dyspnea, anorexia and anuria (<100 mL/d). The patient died of respiratory failure within a week after admission.

## Discussions

Synovial sarcoma is the third most common histological type of soft tissue sarcoma of the extremities, which mainly affects adolescents and young adults [4]. It is slightly more common in men than in women, with a male-to-female ratio of 3:2 [5]. Despite its name, synovial sarcoma does not originate from synovial tissue, but from multipotent stem cells that differentiate into mesenchymal and/or epithelial structures [6]. It is characterized by a unique t(X;18)(p11;q11) chromosomal translocation, which leads to the formation of the SS18-SSX fusion gene [7]. Currently, the best treatment for synovial sarcoma remains undefined, and complete surgical excision of the tumor mass followed by adjuvant radiotherapy and/or chemotherapy is the preferred current choice [8].

Synovial sarcoma often has no specific clinical presentations that distinguish it from other sarcomas. Typically, it presents as a slowly enlarging, deep-seated mass, which is sometimes slightly painful. It is often misdiagnosed as a benign lesion, and simple excision is performed without adequate pre-surgical evaluation. In particular, as the majority of subcutaneous soft-tissue lesions are benign or inflammatory, simple excision is often performed without a biopsy by physicians and surgeons who do not have specific training in oncology [9]. As a result, some aggressive tumors, including synovial sarcoma, can recur within years. Therefore, an early diagnosis is extremely important for the successful treatment of soft tissue synovial sarcoma. Subcutaneous sarcomas have been reported to account for approximately 30 % of all soft tissue sarcomas [9]. In our case, the first clinical sign of synovial sarcoma was a slightly painful subcutaneous mass, and the mass was excised simply. The poor outcome of our patient further stresses the importance of adequate pre-surgical evaluation and post-surgical pathological analysis in the management of a subcutaneous mass.

Although synovial sarcomas are a high-grade soft tissue malignancy, most are characterized by slow growth. The mass may be present for an extended period before presentation and rapid spread and early deaths are seen only occasionally. In the present case, the disease progressed rapidly and the patient died within 4 months of symptom recognition. Given the small tumor size at presentation and the subsequent rapid disease progression, we surmise that local invasion and lymph node metastasis was likely to have occurred before the initial local excision of the mass. Therefore, a wide excision plus adjuvant radiotherapy and/or chemotherapy could have been performed. In addition, for the local excision of a subcutaneous tumor, violation of the underlying fascia may facilitate tumor local invasion, as the fascia provides an excellent deep barrier to tumor spread [9]. Overall, benign tumors should be treated with a marginal excision, whereas malignant tumors should be widely excised. It is important to obtain adequate ablative margins and prevent local recurrence in patients with synovial sarcoma [10].

The development of a non-healing wound that does not respond to conventional antibiotic therapy and local wound care is another interesting point of our case. Although cancer has been identified to be a common cause of non-healing wounds [11], there have been very few reports of patients with synovial sarcoma who developed a non-healing wound. Several reasons may explain the development of a non-healing wound in our patient. Firstly, the sarcoma itself can give rise to wounds. Secondly, severe edema can slow the rate of healing, as dehydration adversely affects optimum wound healing by disturbing cellular metabolism and reducing circulatory blood volume [11,12]. Finally, malnutrition may delay wound healing, as adequate nutrition is essential for effective wound healing [11]. Interestingly, a recent report demonstrated that a postoperative non-healing wound that did not respond to conventional therapy was treated successfully with the local application of 3% citric acid ointment for 25 days in a patient with synovial sarcoma of the knee [13].

MRI is the preferred imaging modality for the evaluation of soft tissue sarcomas because of its excellent tissue contrast and ability to depict the lesion in multiple planes. It is not only useful for evaluating the extent of the tumor and its involvement of adjacent soft-tissue structures, but also for differentiating tumor from muscle tissue and depicting the involvement of neurovascular structures, tendons, fascial/fat planes and bone marrow. On MR images, lesion margins of synovial sarcomas are well defined in 53 to 91% of cases and poorly defined in 9 to 47% [14]. In our case, the lesion was irregular and poorly confined, which was perhaps due to the fact that MRI was performed after local excision and local invasion had occurred. Therefore, preoperative MRI investigations might be more useful for making an early diagnosis of synovial sarcoma. If the findings on preoperative MRI are doubtful, an open or needle biopsy should be performed.

Synovial sarcoma has three major histological subtypes: the biphasic, monophasic and poorly differentiated types [14]. The co-existence of epithelioid cells and spindle cells is a hallmark of the biphasic subtype, while the monophasic subtype is entirely composed of spindle cells [15]. The poorly differentiated subtype is a variant that lacks spindle cells and comprises primitive small round cells. Although there is a strong association between the poorly differentiated subtype and a poor prognosis, this variant of synovial sarcoma shares immunological, ultrastructural and molecular characteristics with the usual form of synovial sarcoma and is often diagnostically challenging [15,16]. Poorly differentiated synovial sarcoma can be further divided into three types: a large cell epithelioid variant, a small cell variant and a high-grade spindle cell variant [15]. In the present case, the presence of numerous small round cells with a high nucleocytoplasmic ratio was seen. This led to the initial diagnosis of a poorly differentiated synovial sarcoma, which was perhaps a small cell variant.

Immunohistochemically, most synovial sarcomas are positive for vimentin, cytokeratin and EMA, but have a lower immunoreactivity for S-100 and CD34 [8]. Although they often co-express both cytokeratin and EMA, only one of them was positive in some cases [17]. Positive staining for CD99 and bcl-2 is also present in many cases of synovial sarcoma [8]. In addition, expression of calretinin, varying from focal to diffuse, is commonly seen in synovial sarcomas [18]. Our case was strongly positive for vimentin and calretinin, moderately positive for CD99 and EMA, and only partially positive for cytokeratin. In addition, cytogenetics also plays an important role in the diagnosis of synovial sarcoma, especially the poorly differentiated subtype, since the reciprocal chromosomal translocation t(X;18) is highly specific for synovial sarcoma; however, this was not performed in our case.

Although curative resection and adjuvant irradiation can improve the local control of synovial sarcoma, metastases eventually develop in most patients. The lung and skeleton are the most commonly affected sites. Regional lymph nodes are involved in 10 to 23% of patients. In our case, although lung metastasis was not detected, pelvic and inguinal lymph node involvement was observed. The pitting edema could be caused by lymphedema due to the presence of lymph node metastases [19]. Patients who develop metastatic soft tissue sarcoma are incurable in most cases, and judicious use of systemic treatment may provide symptom palliation, prevent rapid disease progression and prolong survival. Unfortunately, our patient deteriorated rapidly and died despite systemic therapy.

## Conclusions

In conclusion, we report a case of synovial sarcoma of the buttocks that presented with a non-healing wound and rapid progression after local resection. Despite slow growth in most cases, synovial sarcoma can be fatal within months of recognition if improperly managed. Our case stresses the importance of adequate preoperative evaluation and postoperative pathological analysis in the management of a subcutaneous mass.

## Consent

Written informed consent was obtained from the patient for publication of this case report and any accompanying images.

## Abbreviations

EMA, Epithelial membrane antigen; MRI, Magnetic resonance imaging.

## Competing interests

The authors declare that they have no competing interests.

## Authors’ contributions

ZHY collected the clinical data and performed immunohistochemistry. FY and ZZ collected the clinical data. GG carried out the pathological analysis. ZJS drafted the manuscript. All authors read and approved the final manuscript.
